# Clinical adhesion score (CLAS): development of a novel clinical score for adhesion-related complications in abdominal and pelvic surgery

**DOI:** 10.1007/s00464-020-07621-5

**Published:** 2020-05-14

**Authors:** Elisabeth Jacomine Lier, Barend A. W. van den Beukel, Larsa Gawria, Philip J. van der Wees, Leontine van den Hil, Nicole D. Bouvy, Ying Cheong, Rudy-Leon de Wilde, CLAS Collaboration, Mike Parker, Marc Schreinemacher, Johannes B. C. van der Wal, James W. Fleshman, Malcolm Wilson, Erica Bakkum, Per Lundorff, Michael P. Diamond, Geoffrey Trew, Phillipe Koninckx, Bernhard Krämer, Jasper Verguts, Salomone Di Saverio, Ari Leppaniemi, Michael Sugrue, Yoram Kluger, Ville Sallinen, Mehdi Ouaissi, Luca Ansaloni, David E. Beck, Fausto Catena, Markus Wallwiener, Valerio Mais, Andreas Hackethal, Togas Tulandi, Jakob Burcharth, Jonas Gerner Rasmussen, Yong S. Song, David M. Wiseman, Federico Coccolini, Aurélien Dupre, Jun Kumakiri, Harry van Goor, Martijn W. J. Stommel, Richard P. G. ten Broek

**Affiliations:** 1 Department of Surgery, Radboud University Medical Center, Geert Grooteplein Zuid 10, 6525 GA Nijmegen, The Netherlands; 2 IQ Healthcare, Radboud University Medical Center, Nijmegen, The Netherlands; 3 Department of Surgery, Maastricht University Medical Center, Maastricht, The Netherlands; 4 Department of Surgery, Uniklinik RWTH, Aachen, Germany; 5 Faculty of Medicine, Human Development and Health, University of Southampton, Southampton, UK; 6 Complete Fertility, Princess Anne Hospital, Southampton, UK; 7 Department of Gynecology, Obstetrics and Gynecological Oncology, University Hospital for Gynecology, Pius-Hospital Oldenburg, Oldenburg, Germany

**Keywords:** Adhesions, Adhesion-related complications, Clinical adhesion score

## Abstract

**Background:**

Adhesions are a major cause of long-term postsurgical complications in abdominal and pelvic surgery. Existing adhesion scores primarily measure morphological characteristics of adhesions that do not necessarily correlate with morbidity. The aim of this study was to develop a clinical adhesion score (CLAS) measuring overall clinical morbidity of adhesion-related complications in abdominal and pelvic surgery.

**Methods:**

An international Delphi study was performed to identify relevant score items for adhesion-related complications, including small bowel obstruction, female infertility, chronic abdominal or pelvic pain, and difficulties at reoperation. The CLAS includes clinical outcomes, related to morbidity of adhesions, and weight factors, to correct the outcome scores for the likelihood that symptoms are truly caused by adhesions. In a pilot study, two independent researchers retrospectively scored the CLAS in 51 patients to evaluate inter-observer reliability, by calculating the Intraclass correlation coefficient. During a feasibility assessment, we evaluated whether the CLAS completely covered different clinical scenarios of adhesion-related morbidity.

**Results:**

Three Delphi rounds were performed. 43 experts agreed to participate, 38(88%) completed the first round, and 32 (74%) the third round. Consensus was reached on 83.4% of items. Inter-observer reliability for the CLAS was 0.95 (95% CI 0.91–0.97). During feasibility assessment, six items were included. As a result, the CLAS includes 22 outcomes and 23 weight factors.

**Conclusion:**

The CLAS represents a promising scoring system to measure and monitor the clinical morbidity of adhesion-related complications. Further studies are needed to confirm its utility in clinical practice.

**Electronic supplementary material:**

The online version of this article (10.1007/s00464-020-07621-5) contains supplementary material, which is available to authorized users.

Intra-abdominal adhesions remain the most common cause of long-term complications following abdominal and pelvic surgery despite the broad implementation of minimal invasive surgery [1]. Adhesions develop in 63–97% of patients after abdominal surgical interventions, i.e., general, gynecological, or urological surgery [2–4]. Direct hospital costs associated with complications from adhesions in the USA are estimated at $2.3 billion [5, 6], in Europe costs associated with an episode of small bowel obstruction are approximately €2200 for conservative treatment and €16,000 for surgical treatment [7]. Adhesions are not always symptomatic; however, in many patients, adhesions result in a wide range of complications, occurring months or even many years after surgery. Adhesion-related complications include small bowel obstruction, female infertility, chronic abdominal or pelvic pain, and difficulties at reoperation [1, 3, 8–11].

The overall morbidity of adhesions is difficult to define, because adhesion-related complications are highly heterogeneous. Small bowel obstruction is a surgical emergency that requires hospital admission and conservative or surgical treatment. Approximately 30% of adhesive small bowel obstructions require surgical treatment, making it one of the most frequent indications for emergency surgery [8, 12, 13]. In developed countries, about 60% of abdominal operations are reoperations, in which adhesiolysis is commonly required. The risks and complications of adhesiolysis include (serosal or full thickness) bowel injury, hemorrhage, and conversion from laparoscopy to laparotomy [10, 14, 15]. Adhesions can also impact female fertility. As a result, one in four reproductive aged women seeks fertility treatment after pelvic surgery [3]. Finally, as many as 20% of patients develop chronic abdominal pain after abdominal surgery and adhesions are associated with up to 57% of cases of chronic pain [16–18]. Adhesiolysis for chronic pain remains controversial and most patients are treated conservatively with low success [10].

Measuring adhesion-related morbidity is difficult, since adhesion-related complications develop over a long time period and come with a large variety of physical complaints. The clinical outcome of adhesion-related complications is most relevant for both patients and physicians; however, none of the commonly used adhesion scores measures this aspect. Available adhesion scores, such as Zühlke’s classification [19–21], measure morphological characteristics of adhesions during surgery. Important disadvantages of these scores are that a reoperation is required to assess the severity of adhesions, and that outcomes of these scores have not been validated to correlate with the morbidity of adhesions. This also impedes the interpretation of outcomes of trials on treatment and prevention of adhesions.

The purpose of this study was to develop a clinical adhesion score (CLAS) that measures overall morbidity of intra-abdominal adhesions in postoperative patients following abdominal or pelvic surgery.

## Methods

The development of the CLAS included an international Delphi study, to identify the most relevant score items for the CLAS. A two-step pilot study was conducted to evaluate inter-observer reliability, and to assess if the CLAS covered different clinical scenarios of adhesion- related morbidity, by applying the CLAS to real clinical cases. Ethical approval was not required for this study. A detailed description of the Delphi rounds and data analysis of the Delphi procedure can be found in Supplementary File 2 ‘Methodology’.

### Score items

The CLAS includes outcomes and weight factors. Outcomes are items describing the morbidity or clinical consequences of each adhesion-related complication; small bowel obstruction, difficulties at reoperation, female infertility or subfertility, and chronic abdominal pain. Each outcome corresponds to an outcome score; a score on a scale from 0 to 10, with 0 corresponding to no morbidity and 10 corresponding to very severe morbidity. For example, outcomes of small bowel obstruction could include conservative treatment or surgical treatment, corresponding to an outcome score of 4 and 8, respectively.

The CLAS also includes weight factors: factors that correct an adhesion score for the likelihood that symptoms are truly caused by adhesions, describing the data source on which the judgment is based, i.e., adhesions scored during reoperation, retrospective review of chart, or diagnosis code. Each weight factor includes a corresponding weight factor score on a scale from 0 to 100% (0%: very unlikely that symptoms are caused by adhesions and 100%: a very high likelihood that symptoms are caused by adhesions). These weight factors are added to the CLAS to enable the use of the CLAS without the need of a reoperation to confirm the adhesive etiology of symptoms. Symptoms of adhesion-related complications can be similar to symptoms of other conditions and there is often some uncertainty about the clinical diagnosis of adhesions. Therefore, second-look surgery is considered the gold standard for diagnosis of adhesions. By adding weight factors to the CLAS, the CLAS can be used to score adhesions without a reoperation, since weight factors correct for the uncertainty of a clinical diagnosis.

### Pilot study

In a two-step pilot study, the reliability and feasibility of the CLAS were studied. To assess reliability, two independent researchers (EJL and LG) evaluated the records of a retrospective cohort of patients and provided the ‘clinical adhesion score’ for each case. The inter-observer reliability was measured by calculating the intraclass correlation coefficient (ICC) and percentage of agreement (the number of matches between two observers divided by the total number of scores) for the CLAS scores and the scores for each adhesion-related complication separately. ICC and 95% confident intervals were calculated based on a single measure, absolute-agreement, two-way random-effects model.

For this reliability assessment, we selected patients whose complete medical follow-up was performed at our own institution, from the database of the LAPAD study, a prospective study on adhesiolysis-related morbidity in abdominal surgery. The database included long-term medical follow-up and follow-up in patient-reported questionnaires of 755 patients, including data on small bowel obstruction, complications during reoperation, and chronic pain [15].

A few outcome items of the CLAS were not directly registered in the LAPAD follow-up data, such as the impact of chronic pain on daily life activities. Therefore, a few agreements were made on the interpretation of the LAPAD follow-up before analysis of inter-observer reliability. Second, infertility was not a relevant clinical item among the patients included in the LAPAD study, and the cohort comprised of patients older than the average reproductive age; thus, we decided to exclude fertility from our reliability assessment.

In a second step, we evaluated the feasibility of the CLAS, by assessing whether the CLAS completely covered different clinical scenarios of adhesion-related morbidity in patients of our retrospective cohort. A few case scenarios from outside the LAPAD study, including infertility case scenarios were also considered for assessment of feasibility. The non-consensus items of the final round were re-evaluated in this phase, to see if they would fill any potential shortcomings in the score. A newly proposed item was added to the CLAS if both researchers independently classified that item as relevant for the complete coverage of the adhesion-related morbidity in the clinical scenarios, and if this was in agreement with a small subgroup of the expert panel during a final evaluation of this score.

## Results

### Panelists

43 of 60 international experts who were invited agreed to participate (71.7%). 38 of 43 panelists (88.4%) completed the first round, 31 (72.1%) the second, and 32 (74.42%) the third round. The characteristics of the panelists that completed the first Delphi round are summarized in Table 1.

**Table 1:** Demographics of the panelists that participated in the Delphi study

Demographics of Delphi panelists	
Mean age, year (SD)	49.5 (± 12.2)
Gender (*n* (%))	
Male	34 (89.5%)
Female	4 (10.5%)
Profession (*n* (%))	
Surgeon	22 (57.9%)
Gynecologist	15 (39.5%)
Researcher, not MD	1 (2.6%)
Mean years of experience as MD (Doctor of Medicine) (SD)	22.6 (± 12.0)
Country (*n* (%))	
Belgium	2 (5.3%)
Canada	1 (2.6%)
Denmark	3 (7.9%)
Finland	2 (5.3%)
France	2 (5.3%)
Germany	4 (10.5%)
Ireland	1 (2.6%)
Israel	1 (2.6%)
Italy	5 (13.2%)
Japan	1 (2.6%)
Korea	1 (2.6%)
The Netherlands	7 (18.4%)
United Kingdom	4 (10.5%)
United States of America	4 (10.5%)
Expertise area (*n* (%))	
Adhesions in general	16 (42.11%)
Small bowel obstruction	9 (23.68%)
Difficulties at reoperation	3 (7.90%)
Chronic abdominal pain	5 (13.16%)
Female infertility	5 (13.16%)

### The experts’ opinion

95% of panelists agreed or strongly agreed that it was important that a new adhesion score measuring the morbidity of adhesions was developed. Since over 80% of panelists agreed or strongly agreed that the CLAS should measure the morbidity from small bowel obstruction, difficulties at reoperation, female infertility, and chronic abdominal pain, these complications were all included in the CLAS. 87% of the experts agreed or strongly agreed that the CLAS should include weight factors to correct the adhesion score for the likelihood that symptoms are truly caused by adhesions. Experts agreed that long-term follow-up, with a minimum of 2 years, is required to measure the CLAS reliably. Mean recommended follow-up period by the panel was 3.1 years.

### Delphi study

Prior to the first Delphi round 19 outcomes and 29 weight factors were compiled from the published literature reporting on adhesions and adhesion-related complications.

**Fig. 1:**
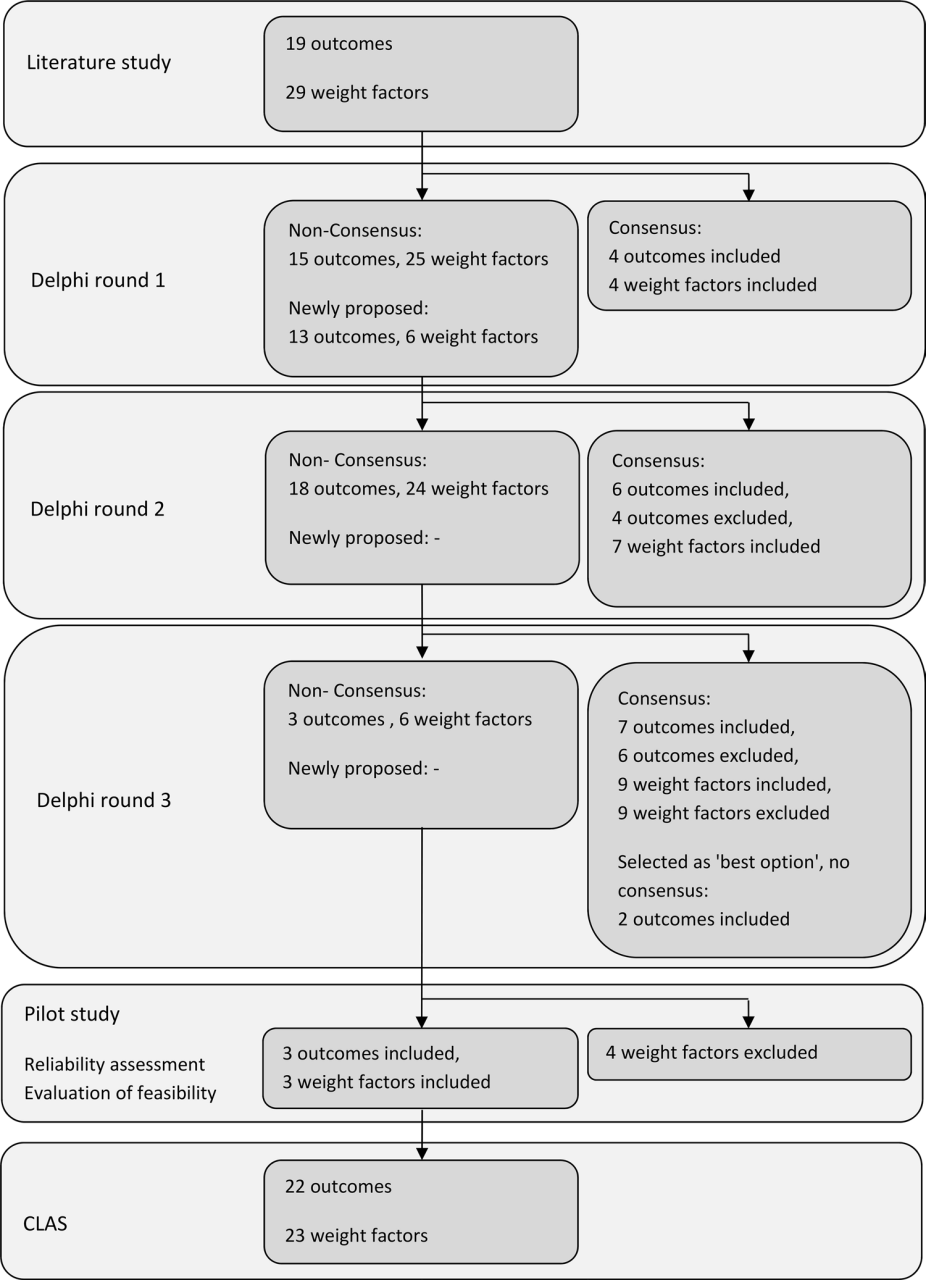
Flowchart of inclusion and exclusion of score items

In the first Delphi round, consensus was reached on four outcomes (included) and four weight factors (included), no items were excluded. In the second round, 13 newly proposed outcomes and six new weight factors were introduced based on the comments of the expert panel in the first round. One outcome and five weight factors were rephrased. Consensus was reached on 10 outcomes (six included, four excluded) and seven weight factors (included). No items were proposed of rephrased for the third round. In Delphi round three, consensus was reached on 13 outcomes (seven included, six excluded) and 18 weight factors (nine included, nine excluded). Two outcomes that did not reach consensus were included, because they were selected by the panel as most appropriate option. Consensus was reached on 83.4% of the items (56/67). After the Delphi procedure, the CLAS included 19 outcomes and 20 weight factors. A flowchart of inclusion and exclusion of score items is shown in Fig. 1. Supplementary File 3 is a summary of score items excluded.

### Pilot study

Reliability was assessed in 51 patients readmitted to the Radboud university medical center for potential postoperative adhesion-related symptoms. We evaluated 30 episodes of small bowel obstruction, 71 reoperations, and 51 postoperative pain assessments in the LAPAD study database and medical records of the study cohort [15].

In 90.1% of assessments there was agreement between both researchers. The ICC for the CLAS was 0.95 (95% confidence interval 0.91–0.97), indicating excellent reliability [22]. The results of the reliability study are summarized in Table 2.

**Table 2:** Reliability analysis of the clinical adhesion score

Reliability analysis clinical adhesion score	Intraclass correlation (95% confidence interval)	% of agreement
Small bowel obstruction	0.90 (0.80–0.95)	96.67
Difficulties at reoperation	0.92 (0.87–0.945)	88.73
Chronic abdominal pain	0.90 (0.84–0.94)	86.27
Sum of assessments	0.92 (0.88–0.94)	89.47
CLAS (*n* = 51)	0.95 (0.91–0.97)	70.59

Sixteen discrepancies in CLAS scores between the researchers were found. One discrepancy in scoring small bowel obstruction was due to different interpretations of the operative report. Discrepancies in scoring difficulties at reoperations (*n* = 8) included different grading in ‘limited adhesiolysis’ and ‘extensive adhesiolysis.’ Discrepancies in chronic pain scored by the researchers (*n* = 7) mainly comprised different grading in percentage of inability due to chronic pain, as derived from patient-reported outcome measures reporting on pain and postoperative function.

During the feasibility assessment, four outcomes and two weight factors were included. One weight factor for chronic pain turned out to be necessary as a weight factor in retrospective cases (in case of clinical diagnosis) and was included in the CLAS (Fig. 1). Three non-consensus outcomes related to infertility were included to provide an appropriate scoring system for infertility, since solely one outcome for infertility was included in the Delphi procedure. Further analysis in the subgroup of gynecologists showed equal consensus rates on the items regarding female infertility, as compared to the whole expert panel. One important non-consensus weight factor excluding major causes of infertility (for example, male factors) was added to the weight factors, to enable the use of the CLAS in clinical cases. During the final evaluation of the score, a weight factor correcting for injuries that are common in specific types of operations was added to the weight factors related to difficulties at reoperations.

### Clinical adhesion score

After the Delphi procedure and the two-step pilot study, the CLAS consisted of 22 outcomes and 23 weight factors, including corresponding outcome scores and weight factor scores. The final version of the CLAS is shown in Table 3. An example of the use of the CLAS in a clinical case is shown in Table 4. A manual for the CLAS is added in Supplementary File 1, including other examples of the use of the CLAS in clinical scenarios.

**Table 3:**
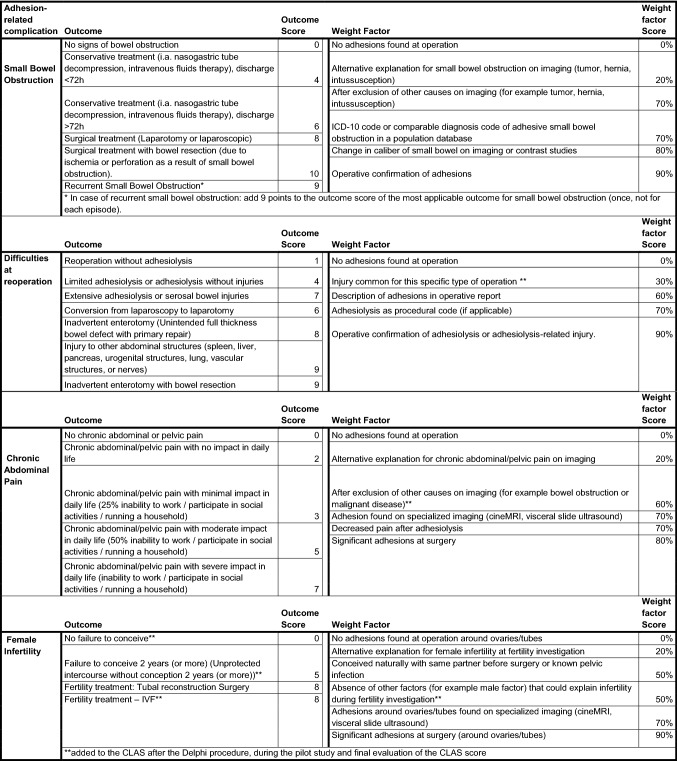
Clinical adhesion score

**Table 4:** Clinical adhesion score of a patient (with a history of abdominal surgery) with a small bowel obstruction which requires surgical treatment

CLAS			
Small bowel obstruction	Surgical treatment (Laparotomy or laparoscopic)	8	7.2
	Operative confirmation of adhesions	90%	
Small bowel obstruction: [Outcome score] × [ Weight Factor Score]			
Difficulties at reoperation	Extensive adhesiolysis or serosal bowel injuries	7	6.3
	Operative confirmation of adhesions	90%	
Difficulties at reoperation: [Outcome score] × [ Weight Factor Score]			
Chronic abdominal pain	–	–	–
Chronic abdominal pain: [Outcome score] × [ Weight Factor Score]			
Female Infertility	–	–	–
Female infertility: [Outcome score] × [ Weight Factor Score]			
Clinical adhesion score (+)			13.5

There is an operative confirmation of adhesive small bowel obstruction. Furthermore, extensive adhesiolysis was performed to enter the abdominal cavity

## Discussion

The CLAS is the first score that assesses adhesions by their clinical impact on morbidity, which enables a complete evaluation of the consequences of adhesion-related complications. Clinical outcome measures used in previous research on adhesions are often based on readmission for small bowel obstruction and to smaller extent complications at reoperations. Chronic abdominal pain and female infertility are often not evaluated in research into adhesions and adhesion barriers, leading to an incomplete assessment of adhesion-related morbidity [23]. However, chronic pain is one of the most frequent adhesion-related complications and of major relevance from a patients’ perspective [24, 25]. Another drawback of powering a trial on small bowel obstruction alone is its relatively low incidence. Thus, studies with small bowel obstruction as an outcome require a very large sample size which is often not feasible.

Most existing adhesion scores, measuring morphology and distribution of adhesions, require surgery to determine the adhesion score, and their correlation with adhesion-related morbidity has not been validated [19–21, 26]. The need to perform a repeat surgery for adhesion evaluation limits their use to only a small number of surgical and gynecological procedures, e.g., two steps of surgery. Traditionally, fertility surgery and loop ileostomy closure have been the most commonly used models for clinical studies on adhesion formation [23]. With the decline in second-look procedures after reproductive surgery, recently cesarean section has also been popularized as a model for adhesion prevention study. Cesarean section, however, has a low risk of adhesion-related morbidity [27, 28].

The CLAS measures morbidity and does not require reoperation for score measures, waiving ethical issues concerning the use of second-look procedures and avoiding potential new adhesion formation from second-look surgery. This also opens the possibility to design new adhesion prevention trials in procedures which are at high risk of adhesion-related complications, instead of procedures with a planned reoperation or high reoperation rate. From a health technology assessment perspective, colorectal, abdominal wall, and oncological gynecological operations are expected to have much more benefit from adhesion prevention and it would be preferable if new trials are designed for those patient groups [3, 23, 28]. Furthermore, most published reports on adhesion prevention only measured complications of adhesions that are managed within the same surgical specialty. In daily practice, patients with adhesions often develop complications that are managed by surgical specialties other than the specialty performing the initial operation. The CLAS enables measurement of the full spectrum of adhesion-related morbidity, by different surgical specialties, including outcomes on chronic pain and female infertility [23, 29].

The CLAS has been developed through the Delphi method. This anonymous consensus method prevents influence of reputation or personality of participants in the development of the CLAS, thus minimizing conflicts of interest related to specialization or subjective opinion. The expert panel included both surgeons and gynecologists specialized in intra-abdominal adhesions in general or in one specific adhesion-related complication, contributing to a reliable assessment of all proposed score items. A high number of participants completed all Delphi rounds (> 70%). The CLAS has been developed as a clinical outcome, and therefore, although patient representatives were involved in the study process, they were not part of the Delphi panel.

The CLAS showed excellent reliability in ICC analysis in both the CLAS score in general and the separately scored complications included in the CLAS. Although not all necessary data for the CLAS were available in our retrospective cohort, the LAPAD study database provided reliable data on morbidity of adhesions and adhesion-related complications from both medical records and observations by independent researchers. Most of the discrepancies were small differences in ratings, for example, the rating of the severity of adhesiolysis in reoperations, or the percentage impact of chronic pain in daily life. The discrepancies could be largely explained by differences in the interpretation of operative reports and patient-reported questionnaires, in which reporting on these complications was brief or not specific.

An important implication of the CLAS is the scoring of the weight factors. These weight factors were introduced as measurable criteria for the likelihood of complications to be caused by adhesions. When more individual patient details are available, clinicians might intuitively estimate the likelihood differently on an individual basis. Still, we would recommend adhering to these weight factors, because they have been designed to comprise most common data sources and the percentages are based on those provided by epidemiological evidence. Further, almost all weight factors include categories for confirmation or rejection of the diagnosis of adhesion-related complication by operative findings in the individual patient. Because the CLAS score is a clinical score, practice variation can impact individual scores and weight factors. When designing a study, practice variation should be counted for either by taking this variation into account when calculating the necessary sample size analysis or by standardizing follow-up and care by adhering to guidelines for adhesion-related complications. In this way, clinical decision-making for adhesion-related complications is standardized as much as possible.

When using the CLAS score in the design of a clinical trials it is important to account for gender specific aspects of adhesions, especially in cohorts including female patients at a younger age. Female patients can receive extra points in the fertility domain, which is not applicable in men. Apart from infertility, epidemiological studies have reported a higher risk for adhesion-related complications in women [1, 30]. When designing or reporting an adhesion–related study, it is important to account for patient sex and stratify randomization for sex (see Supplementary File 1: Manual).

There are some limitations to this study that need to be discussed. During the first Delphi round, > 80% of the experts agreed or strongly agreed that chronic pain and infertility should be measured in the CLAS. However, during the following rounds, the Delphi method could not fully resolve controversies on these complications; instead of more consensus towards inclusion or exclusion in further rounds, the results showed comparable consensus rates in each Delphi round, even in the subgroup analysis of gynecologists. The 9-point Likert scale (with only 1 and 9 labeled verbally) was used to enable a rating matching the uncertainty and discussion on adhesion-related complications; however, this scale might have led to a wider distribution of the expert’s opinion or a more different interpretation of the categories. Probably, a 5-point scale would have obliged participants to make a clear choice and could have led to higher consensus rates on some of the score items. Since some aspects of chronic abdominal pain and female infertility are often debated by adhesion experts, we did not expect to obtain full consensus on all items, and therefore, we predefined the number of Delphi rounds. Although the items for chronic pain and female infertility added in the feasibility study did not reach consensus for inclusion, we included these items to enable the use of a complete scoring system in retrospective cases and for monitoring quality of care.

There are also some limitations on the CLAS score itself. First, the weight factors included by the panelists were mainly related to surgery or (specific) imaging techniques, which indicate the difficulty to clinically diagnose other causes of infertility and pain. When the CLAS is used in prospective studies or as part of the design of an adhesion prevention trial, the use of specific diagnostic tests for adhesions is recommended. The weight factors about specific diagnostic instruments might also be a limitation in the implementation of this score, since these specific tests are not always available to diagnose adhesions or to exclude other causes of symptoms. Furthermore, the impact of the results of these tests, such as adhesions found on specialized imaging, is limited in current clinical practice. However, the CLAS also offers possibilities to be used without specialized imaging, since the score also includes outcomes and weight factors corresponding to conventional imaging or conservative and surgical treatment modalities.

One of the challenges in development of this score was defining the morbidity score for different outcomes. Although it is possible to relate scores to existing literature on morbidity or PROMs related to one specific adhesion-related complication, literature directly comparing morbidity between different subgroups of adhesion-related complications is lacking. To reach a balanced scoring, all panel members received relevant literature references for all complications, and the panel included experts on all four types of complications defined.

The CLAS does require a longer period of follow-up compared with morphological adhesion scores. There is often a lag time between the development of adhesions and the occurrence of adhesion-related complications such as a small bowel obstruction. New cases of adhesion-related complications continue to develop for many years after surgery, although approximately 70% develops within the first two years after surgery [1, 3, 8]. This is consistent with the minimal postoperative follow-up of 24 months and recommended follow-up of 36 months as advised by the Delphi panel.

Finally, this study included the development of the CLAS and some first steps in the validation of the CLAS, in a small number of patients from a retrospective cohort. Further evaluation of reliability and validation in prospective cohorts is necessary, for example, studies in multicenter cohorts or with investigators from different medical centers or surgical specialties. Since fertility was not taken into account in our pilot study, this also includes assessments in patient groups with female, fertile aged patients, and female patients with fertility-related morbidity as a result of adhesions. We did not evaluate fertility in our cohort, since these data were not available, e.g., the patients in our cohort were older than the average reproductive age, and infertility was not a relevant clinical item among the patients included in the LAPAD study. Further development and improvement could also be expected when the CLAS is used by other researchers and physicians, to evaluate its feasibility in various types of clinical study designs and to confirm its utility in clinical practice.

The CLAS has the potential to fill an important gap in current adhesion research and in clinical patient care. The lack of clinical outcome data is one of the main barriers in the implementation of adhesion prevention strategies, and adhesion barriers specifically [23, 31, 32]. The CLAS can be integrated into the design of new studies investigating adhesion-related complications and trials for novel adhesion prevention to provide evidence for efficacy on clinically relevant outcomes from a medical perspective. The CLAS can additionally serve as a clinical tool to evaluate the effect of treatment, or as a decision-making tool in treatment strategies. An important advantage of the CLAS is the possibility to measure the full spectrum of adhesion-related morbidity, both prospectively and retrospectively. Furthermore, the CLAS can be used in patients with or without a reoperation to assess adhesions and in patients who have or have not had special imaging to diagnose adhesions. Our group also took the initiative to develop a tool measuring the consequences of adhesions from the patient’s perspective. This patient-reported outcome measure (PROM) will score physical and mental symptoms of small bowel obstruction, chronic abdominal pain, and female infertility, and measures their influence on functional status in patients.

## Conclusion

The CLAS is a scoring system to measure and monitor the morbidity of intra-abdominal adhesions after abdominal and pelvic surgery, by scoring the clinical consequences of adhesion-related complications: small bowel obstruction, difficulties at reoperation, chronic abdominal pain, and female sub- and infertility. The CLAS could enable the evaluation of both surgical and gynecological outcomes related to adhesion formation in patient care and may help to evaluate the efficacy of adhesion prevention and treatment in future trials. Further studies are needed to confirm its validity and utility in clinical practice.

## Electronic supplementary material

Below is the link to the electronic supplementary material.

Supplementary file1 (PDF 127 kb)

Supplementary file2 (PDF 137 kb)

Supplementary file3 (PDF 143 kb)
