# Supplementary material for: Clinical adhesion score (CLAS): development of a novel clinical score for adhesion-related complications in abdominal and pelvic surgery
Source: Surg Endosc. 2020 May 14;35(5):2159–68. doi: 10.1007/s00464-020-07621-5 (PMC8057995; doi:10.1007/s00464-020-07621-5)
Supplement: Supplementary file 1 — Supplementary file1 (PDF 127 kb) [file 464_2020_7621_MOESM1_ESM.pdf]

Supplementary File 1: Manual of the Clinical Adhesion Score(CLAS)

General instructions

Introduction

The Clinical Adhesion Score (CLAS) is an adhesion score measuring the morbidity of intra-abdominal adhesions and adhesion-related complications in patients who have had any type of intraperitoneal surgery. The morbidity of adhesions is defined by the clinical consequences of the following adhesion-related complications: small bowel obstruction, difficulties at reoperation, female infertility, and chronic abdominal/pelvic pain. The CLAS does not score morphological characteristics of adhesions during reoperations.

Definitions used in the CLAS

* Small Bowel Obstruction: interruption of the normal flow of intraluminal content of the small intestines.

* Difficulties at reoperation: Complications or difficulties during repeat intraperitoneal surgery (general, gynecological, vascular, and urological surgery).

* Female infertility: inability to become pregnant or the inability to carry a pregnancy to a live birth following either a previous pregnancy or a previous ability to carry a pregnancy to a live birth after 12 months or more of unprotected intercourse (female *secondary* infertility).

* Chronic abdominal pain: continuous or intermittent abdominal/pelvic discomfort lasting for at least six months.

CLAS items

1. Outcomes

Items describing the clinical consequences or morbidity of adhesion-related complications, listed for each adhesion-related complication (small bowel obstruction, difficulties at reoperation, female infertility, and chronic abdominal/pelvic pain).

2. Outcome score

A score on a scale from 0 to 10 corresponding to the severity of each outcome (see 1. Outcomes) with 0 corresponding to no morbidity and 10 with very severe morbidity.

3. Weight factors

Weight factors are introduced because the diagnosis of adhesions is sometimes unsure: it might be difficult to determine whether symptoms are truly caused by adhesions without a reoperation to confirm adhesive etiology. Weight factors are factors to correct an adhesion morbidity score for the likelihood that symptoms are caused by adhesions, describing the data source i.e. adhesions scored during reoperation, retrospective review of chart, or diagnosis code (specific weight factors for each adhesion-related complication).

4. Weight factor score

A score on a scale from 0-100%, corresponding to the likelihood of adhesive etiology.

Mean recommended time of follow-up

A minimal follow-up of two years is required to reliable assess the CLAS, since there is often a lag time between the development of adhesions and the occurrence of adhesion-related complications such as a small bowel obstruction. New cases of adhesion-related complications continue to develop for many years after surgery, although approximately 70% develops within the first two years after surgery. This is consistent with the minimal postoperative follow-up of 24 months and recommended follow-up of 36 months as advised by the Delphi panel.

Controlling for sex

It is advised to control for sex when using the CLAS and when designing a trial on adhesion prevention for two reasons. First by nature of the score , female patients may accrue additional points on the scale in the fertility domain. Second epidemiological studies report a higher risk for

adhesion-related complications in women in general (see for example Krielen et all, 2020, and Nunoo-Mensah et all, 2009).

Controlling for gender in prospective research cohorts can be performed by stratifying for gender.

In retrospective study cohorts, it is recommended to analyze the influence of gendersex, and to report the results for men and women separately.

How to use the CLAS:

1. Select the adhesion-related complication: small bowel obstruction, difficulties at reoperation, chronic abdominal pain or female infertility.
2. Check the outcomes for the complication of interest and select the outcome that is applicable for the patient or study case report form.

   If more than one outcome is applicable (in one episode of symptoms), please choose the outcome corresponding to the highest morbidity rate (=outcome score) for that specific complication.
3. Fill in the outcome score corresponding to the specific outcome (see the table below)
4. Check the weight factors for the complication(s) of interest and select the weight factor that is applicable for the patient or study case report form; thus, in which way are the adhesions diagnosed and if not, which weight factor is most appropriate for the patient's case.

   If more than one weight factor is applicable (in one episode of symptoms), please choose the weight factor with the highest corresponding weight factor score for that specific complication.
5. Fill in the weight score corresponding to the chosen weight factor.
6. The score for each adhesion-related complication: [Outcome score] x [Weight Factor Score]. In case of recurrent clinical episodes for the same class of complications in the follow-up period of one patient, please include the score of the episode with the highest score. For recurrent episodes of small bowel obstruction, an additional outcome has been added to the CLAS.
7. Clinical adhesion score: sum of the separate scores of each adhesion-related complication.

**CLAS**			
**Small Bowel Obstruction**	[Outcome]	[Outcome score]	
	[Weight Factor]	[Weight Factor Score]	
Small Bowel Obstruction: [Outcome score] x [Weight Factor Score]			
**Difficulties at reoperation**	[Outcome]	[Outcome score]	
	[Weight Factor]	[Weight Factor Score]	
Difficulties at reoperation: [Outcome score] x [Weight Factor Score]			
**Chronic abdominal pain**	[Outcome]	[Outcome score]	
	[Weight Factor]	[Weight Factor Score]	
Chronic abdominal pain: [Outcome score] x [Weight Factor Score]			
**Female Infertility**	[Outcome]	[Outcome score]	
	[Weight Factor]	[Weight Factor Score]	
Female Infertility: [Outcome score] x [Weight Factor Score]			
**Clinical adhesion score (+)**			

Example 1: Clinical Adhesion score of a patient (with a history of abdominal surgery) suffering from a small bowel obstruction (diagnosed on imaging), requiring conservative treatment and hospital stay for more than 72 hours.

**CLAS**			
**Small Bowel Obstruction**	Conservative treatment(i.a. nasogastric tube decompression, intravenous fluids therapy), discharge >72h	6	
	Change in caliber of small bowel on imaging or contrast studies	80%	
Small Bowel Obstruction: [Outcome score] x [Weight Factor Score]			4.8
**Difficulties at reoperation**			
Difficulties at reoperation: [Outcome score] x [Weight Factor Score]			-
**Chronic abdominal pain**			
Chronic abdominal pain: [Outcome score] x [Weight Factor Score]			-
**Female Infertility**			
Female Infertility: [Outcome score] x [Weight Factor Score]			-
**Clinical adhesion score (+)**			4.8

Example 2: Clinical Adhesion score of a patient (with a history of abdominal surgery) with a small bowel obstruction which requires surgical treatment. There is an operative confirmation of adhesive small bowel obstruction. Furthermore, extensive adhesiolysis was performed to enter the abdominal cavity.

**CLAS**			
**Small Bowel Obstruction**	Surgical treatment (Laparotomy or laparoscopic)	8	
	Operative confirmation of adhesions	90%	7.2
Small Bowel Obstruction: [Outcome score] x [Weight Factor Score]			
**Difficulties at reoperation**	Extensive adhesiolysis or serosal bowel injuries	7	
	Operative confirmation of adhesions	90%	
Difficulties at reoperation: [Outcome score] x [Weight Factor Score]			6.3
**Chronic abdominal pain**			
Chronic abdominal pain: [Outcome score] x [Weight Factor Score]			-
**Female Infertility**			
Female Infertility: [Outcome score] x [Weight Factor Score]			-
**Clinical adhesion score (+)**			13.5

Example 3: Clinical Adhesion score of a patient with a history of abdominal operations and recurrent small bowel obstruction, diagnosed with a small bowel obstruction. After 36 hours of conservative treatment, surgical treatment, including a resection of ischemic bowel parts is performed. There is an operative confirmation of adhesive etiology. The surgery also includes a conversion from laparoscopy to laparotomy due to adhesion-related difficulties.

**CLAS**			
**Small Bowel Obstruction**	Surgical treatment with bowel resection (due to ischemia or perforation as a result of small bowel obstruction).<br>+<br>Recurrent Small Bowel Obstruction	19 (10+9)	
	Operative confirmation of adhesions	90%	
	Small Bowel Obstruction: [Outcome score] x [Weight Factor Score]		17.1
**Difficulties at reoperation**	Conversion from laparoscopy to laparotomy	6	
	Operative confirmation of adhesions	90%	
	Difficulties at reoperation: [Outcome score] x [Weight Factor Score]		5.4
**Chronic abdominal pain**			
	Chronic abdominal pain: [Outcome score] x [Weight Factor Score]		-
**Female Infertility**			
	Female Infertility: [Outcome score] x [Weight Factor Score]		-
	**Clinical adhesion score (+)**		22.5

Example 4: Clinical Adhesion score, maximum score

**CLAS**			
**Small Bowel Obstruction**	Surgical treatment with bowel resection (due to ischemia or perforation as a result of small bowel obstruction).<br>+<br>Recurrent Small Bowel Obstruction	19 (10+9)	
	Operative confirmation of adhesions	90%	
	Small Bowel Obstruction: [Outcome score] x [Weight Factor Score]		17.1
**Difficulties at reoperation**	Inadvertent enterotomy with bowel resection	9	
	Operative confirmation of adhesions	90%	
	Difficulties at reoperation: [Outcome score] x [Weight Factor Score]		8.1
**Chronic abdominal pain**	Chronic abdominal/pelvic pain with severe impact in daily life (inability to work / participate in social activities / running a household)	7	
	Significant adhesions at surgery	80%	5.6
	Chronic abdominal pain: [Outcome score] x [Weight Factor Score]		
**Female Infertility**	Fertility treatment – IVF	8	
	Significant adhesions at surgery (around ovaries/tubes)	90%	
	Female Infertility: [Outcome score] x [Weight Factor Score]		7.2
	**Clinical adhesion score (+)**		38
