# Supplementary material for: Clinical adhesion score (CLAS): development of a novel clinical score for adhesion-related complications in abdominal and pelvic surgery
Source: Surg Endosc. 2020 May 14;35(5):2159–68. doi: 10.1007/s00464-020-07621-5 (PMC8057995; doi:10.1007/s00464-020-07621-5)
Supplement: Supplementary file 2 — Supplementary file2 (PDF 137 kb) [file 464_2020_7621_MOESM2_ESM.pdf]

Supplementary File 2: Methodology CLAS

Delphi study

The Delphi method is a technique to acquire consensus on the opinions of experts through a series of anonymous questionnaires. Results from each questionnaire are summarized and shared with the panelists of the expert panel, who are asked to evaluate the survey items again until consensus has been reached or until the predefined number of rounds has been performed. Additional questions can be added to the following rounds, based upon suggestions of the panelists during the previous round. (1, 2)

The Delphi procedure in this study included three rounds, during which the panelists rated the appropriateness of the proposed score items to identify the most relevant score items for the CLAS. The panelists were also asked to fill in their demographic characteristics and to give their opinion on the novel adhesion score (The questionnaire can be found at the end of this methodology section).

Expert panel

Participants in the expert panel were authors who have made significant contributions to the literature on adhesions, with at least one article on adhesion-related complications or adhesion prevention published in a core medical journal in the preceding 10 years as a senior author or principal investigator (in PubMed up to May 2017), or were part of a networks of physicians who have participated in guideline committees for adhesion-related complications. We compiled an international expert group of general surgeons and gynecologists, with a mixture of experts specialized in a specific adhesion-related complication or adhesions in general. The characteristics of the panelists that completed the first Delphi round are summarized in Table 1. Names and expertise area of involved experts are listed below.

Supp. File Table 1: Names of experts involved in the Delphi panel, who have completed the first Delphi round.

Panelist	Profession	Expertise	Panelist	Profession	Expertise
L Ansaloni	Surgeon	Small Bowel Obstruction	A Leppaniemi	Surgeon	Small Bowel Obstruction
E Bakkum	Gynecologist	Adhesions in general	P Lundorff	Gynecologist	Adhesions in general
DE Beck	Surgeon	Small Bowel Obstruction	V Mais	Gynecologist	Female infertility
ND Bouvy	Surgeon	Adhesions in general	M Ouaissi	Surgeon	Small Bowel Obstruction
RPG ten Broek	Surgeon	Adhesions in general	M Parker	Surgeon	Adhesions in general
J Burcharth	Surgeon	Chronic abdominal pain	V Sallinen	Surgeon	Small Bowel Obstruction
F Catena	Surgeon	Small Bowel Obstruction	S Di Saverio	Surgeon	Small Bowel Obstruction
Y Cheong	Gynecologist	Chronic abdominal pain	M Schreinemacher	Surgeon	Adhesions in general
F Coccolini	Surgeon	Difficulties at reoperation	YS Song	Gynecologist	Chronic abdominal pain
MP Diamond	Gynecologist	Adhesions in general	MWJ Stommel	Surgeon	Adhesions in general
A Dupre	Surgeon	Difficulties at reoperation	M Sugrue	Surgeon	Small Bowel Obstruction
JW Fleshman	Surgeon	Adhesions in general	G Trew	Gynecologist	Adhesions in general
J Gerner	Surgeon	Chronic abdominal pain	T Tulandi	Gynecologist	Female infertility
H van Goor	Surgeon	Adhesions in general	J Verguts	Gynecologist	Adhesions in general
A Hackethal	Gynecologist	Female infertility	JBC van der Wal	Surgeon	Adhesions in general
Y Kluger	Surgeon	Small Bowel Obstruction	M Wallwiener	Gynecologist	Female infertility
P Koninckx	Gynecologist	Adhesions in general	RL de Wilde	Gynecologist	Female infertility
B Krämer	Gynecologist	Adhesions in general	M Wilson	Surgeon	Adhesions in general
J Kumakiri	Gynecologist	Difficulties at reoperation	DM Wiseman	Researcher	Chronic abdominal pain

Compilation of the concept score items of the CLAS

The score items that were evaluated during the first Delphi round, were compiled from published literature on adhesions and adhesion-related complications, or were items based on the definitions of the adhesion-related complications that are included in the CLAS. PubMed was searched for relevant outcomes and weight factors, using the search terms for morbidity, adhesions, small bowel obstruction, difficulties at reoperation, chronic abdominal pain, and female infertility (articles from January 1990 to April 2017). Additional references were provided by a manual search of reference lists and by the expert panel.

Small bowel obstruction was defined as an interruption of the normal flow of intraluminal content of the small intestines. Difficulties at reoperation included complications or difficulties during repeat abdominal surgery (general, gynecological, vascular or urological surgery). Adhesion-related female (secondary) infertility was defined as inability to become pregnant or the inability to carry a pregnancy to a live birth following either a previous pregnancy or a previous ability to carry a pregnancy to a live birth after 12 months or more of unprotected intercourse, following abdominal

surgery. Chronic abdominal pain is defined as continuous or intermittent abdominal/pelvic discomfort lasting for at least six months after surgery. Adherence to IASP guidelines for diagnosis of chronic pain is recommended. An overview of the search terms and background literature of the CLAS can be found below.

Delphi rounds

Experts were invited by email to participate in the Delphi study. The experts who agreed to participate, received an email including a personal link to the online survey, a manual for the first Delphi round, and recent evidence of research into adhesions which was used to compile the items of the CLAS.

Each Delphi round consisted of an online questionnaire, developed in Castor EDC (Castor Electronic Data Capture, Ciwit BV Amsterdam, The Netherlands 2017).

In the first Delphi round questionnaire, the experts were asked if the proposed outcomes and weight factors were appropriate for a specific adhesion-related complication in a clinical adhesion score. The appropriateness of both outcomes and weight factors were rated on a 9-point Likert scale ranging from 1 to 9, with 1 as very inappropriate to 9 as very appropriate. Panelists were asked to determine the severity of each outcome on a numeric rating scale from 0-10 as 'outcome score'. The corresponding weight factor score for each weight factor (likelihood of adhesive etiology) was rated on a scale from 0-100%. Panelists were invited to add a comment or suggestion on every survey question and to propose new score items. The panelists were also asked to fill in their demographic characteristics and to give their opinion on eleven statements about the development and content of the novel adhesion score, rated on a 5-point Likert scale (see below).

The panelists who completed the first round were invited to participate in further rounds. The panelists received a summary of the results of the previous round with a group mean score, median score, and percentage of agreement (percentage of panelists that rated the appropriateness as 7-9) for each outcome and weight factor.

In the second and third round the items that did not reach consensus in the former round were

reevaluated on their appropriateness. Furthermore, the panelists were asked to rate the appropriateness of newly proposed items and to determine the corresponding outcome or weight factor score for these items. If an outcome or weight factor was rephrased or redefined, the original item and the alternative proposed were both rated on the 9-point rating scale of appropriateness. In addition, in separate questions we asked whether the original item or the alternative proposed was the most appropriate option. Again, panelists had the opportunity to add comments on every survey question and were asked to add a recommended time of follow-up for the CLAS. We predefined a maximum of 3 Delphi rounds because selection of the relevant CLAS items was not to be expected in further rounds. In each round, panelists were given 17 days to complete the questionnaire and two email reminders were sent to non-responders.

Analysis of the Delphi data

Data analysis was performed using IBM SPSS statistics v22.0. Only completed questionnaires were analyzed. In the first rounds, consensus was defined as ≥70% of panelists scoring an outcome or weight factor as 1-3 or 7-9. Items were included if ≥70% of participants scored an item as 7-9, and <15% scored it as 1-3 on the 9-point rating scale of appropriateness. Items were excluded if ≥70% of participants scored an item as 1-3 and <15% scored it as 7-9. Items that did not meet these criteria were classified as 'non-consensus' items and were reevaluated in the subsequent Delphi round.

In the final Delphi round, items were included if >60% of the panelists scored an item as 7-9. Items were excluded if <50% of the participants scored an item as 6-9. If several outcomes for comparable clinical scenarios were included, the option that was rated as 'most appropriate option' was selected and included. The comments and suggestions from the panelists were evaluated individually after each Delphi round. Newly proposed outcomes and weight factors were added to the Delphi procedure, if suggested by two or more panel members. Furthermore, several items were rephrased based on comments made by panel members.

The outcome scores and weight factor scores were determined as the mean of mode, median and

mean score as rated by the expert panel.

Search terms concept items of the CLAS.

Intestinal obstruction[mesh] OR "bowel obstruction" [tiab] OR SBO[tiab] OR infertility, female[mesh] OR infertility[tiab] OR enterotomy[tiab] OR abdominal pain[mesh] OR pelvic pain[mesh] OR "abdominal pain" [tiab] OR "pelvic pain" [tiab] OR intestinal disease/surgery[mesh] OR abdomen/surgery[mesh] OR peritoneum/surgery[mesh] OR Laparoscopy[mesh] OR laparotomy[mesh] OR laparo*[tiab]

AND

Tissue adhesions[mesh] OR adhes*[tiab]) AND (abdo*[tiab] OR abdomen[mesh] OR pelvis[mesh] OR pelvi*[tiab] OR periton*[tiab] OR Peritoneum[mesh] OR Laparoscopy[mesh] OR laparotomy[mesh] OR laparo*[tiab] OR intestine[mesh] OR intestin*[tiab]

Limits*:* Subheadings: NOT (animal NOT human). Publication date: 1 January 1990 or later

*[mesh]=medical subheading, controlled vocabulary as used by National Library of Medicine for indexing articles. [tiab]=word in title or abstract. *=truncation; retrieves all possible suffix variations of root word indicated*

Background Literature Clinical Adhesion Score (CLAS)

1. Adhesions in general

- ten Broek RP, Issa Y, van Santbrink EJ, Bouvy ND, Kruitwagen RF, Jeekel J, et al. Burden of adhesions in abdominal and pelvic surgery: systematic review and met-analysis. BMJ. 2013;347:f5588.

- Bolnick A, Bolnick J, Diamond MP. Postoperative adhesions as a consequence of pelvic surgery. J Minim Invasive Gynecol. 2015;22(4):549-63

- ten Broek RP, Stommel MW, Strik C, van Laarhoven CJ, Keus F, van Goor H. Benefits and harms of adhesion barriers for abdominal surgery: a systematic review and meta-analysis. Lancet. 2014;383(9911):48-59.

2. Adhesive small bowel obstruction

- Parikh JA, Ko CY, Maggard MA, Zingmond DS. What is the rate of small bowel obstruction after colectomy? Am Surg. 2008;74(10):1001-5.

- Catena F, Di Saverio S, Coccolini F, Ansaloni L, De Simone B, Sartelli M, et al. Adhesive small bowel adhesions obstruction: Evolutions in diagnosis, management and prevention. World J Gastrointest Surg. 2016;8(3):222-31.

- Catena F, Ansaloni L, Di Saverio S, Pinna AD, World Society of Emergency S. P.O.P.A. study: prevention of postoperative abdominal adhesions by icodextrin 4% solution after laparotomy for adhesive small bowel obstruction. A prospective randomized controlled trial. J Gastrointest Surg. 2012;16(2):382-8.

3. Difficulties during reoperation

- ten Broek RP, Strik C, Issa Y, Bleichrodt RP, van Goor H. Adhesiolysis-related morbidity in abdominal surgery. Ann Surg. 2013;258(1):98-106.

- Kumakiri J, Kikuchi I, Kitade M, Kuroda K, Matsuoka S, Tokita S, et al. Incidence of complications during gynecologic laparoscopic surgery in patients after previous laparotomy. J Minim Invasive Gynecol. 2010;17(4):480-6.
- Dupre A, Lefranc A, Buc E, Delpero JR, Quenet F, Passot G, et al. Use of bioresorbable membranes to reduce abdominal and perihepatic adhesions in 2-stage hepatectomy of liver metastases from colorectal cancer: results of a prospective, randomized controlled phase II trial. Annals of surgery. 2013;258(1):30-6.

4. Female Infertility

- Gorgun E, Remzi FH, Goldberg JM, Thornton J, Bast J, Hull TL, et al. Fertility is reduced after restorative proctocolectomy with ileal pouch anal anastomosis: a study of 300 patients. Surgery. 2004;136(4):795-803.
- Hahnloser D, Pemberton JH, Wolff BG, Larson D, Harrington J, Farouk R, et al. Pregnancy and delivery before and after ileal pouch-anal anastomosis for inflammatory bowel disease: immediate and long-term consequences and outcomes. Dis Colon Rectum. 2004;47(7):1127-35.

5. Chronic Abdominal Pain

- van den Beukel BA, de Ree R, van Leuven S, Bakkum EA, Strik C, van Goor H, et al. Surgical treatment of adhesion-related chronic abdominal and pelvic pain after gynecological and general surgery: a systematic review and meta-analysis. Hum Reprod Update. 2017:1-13.
- Cheong YC, Reading I, Bailey S, Sadek K, Ledger W, Li TC. Should women with chronic pelvic pain have adhesiolysis? BMC Womens Health. 2014;14(1):36.
- Swank DJ, Swank-Bordewijk SCG, Hop WCJ, van Erp WFM, Janssen IMC, Bonjer HJ, et al. Laparoscopic adhesiolysis in patients with chronic abdominal pain: a blinded randomised controlled multi-centre trial. Lancet. 2003;361(9365):1247-51.

Questionnaire 'The expert's opinion'

Statements were rated on a 5-point Likert scale ranging from 1 to 5, with 1 as Strongly disagree to 5 as Strongly agree.

Statement

1. Existing adhesion scores lack a correlation with clinical outcomes and morbidity
2. Existing adhesion scores are of limited value in the design of clinical trials on adhesion prevention
3. Existing adhesion scores are of limited value to monitor outcomes of surgery related to adhesions
4. It is important that a new adhesion score measuring morbidity of adhesions is developed
5. A new clinical adhesion score measuring the morbidity of adhesion-related complications is a valuable addition to research into adhesions
6. A new clinical adhesion score measuring the morbidity of adhesion-related complications is a valuable addition to treatment of adhesions in clinical practice.
7. It is important that a new adhesion score measures morbidity from adhesive small bowel obstruction
8. It is important that a new adhesion score measures morbidity from difficulties at reoperation
9. It is important that a new adhesion score measures morbidity from chronic abdominal/pelvic pain
10. It is important that a new adhesion score measures morbidity from female infertility
11. Diagnosis of adhesions is sometimes unsure. It is important to correct an adhesion morbidity score for the likelihood that symptoms are truly caused by adhesions

References

1. Graham B, Regehr G, Wright JG. Delphi as a method to establish consensus for diagnostic criteria. J Clin Epidemiol. 2003;56(12):1150-6.
2. Fitch K B, S., Aguilar, M., Burnand, B., & LaCalle, J. . The RAND/UCLA Appropriateness Method User's Manual. 1st ed. Rand Corporation Santa Monica. 2001.
