# Supplementary material for: Clinical adhesion score (CLAS): development of a novel clinical score for adhesion-related complications in abdominal and pelvic surgery
Source: Surg Endosc. 2020 May 14;35(5):2159–68. doi: 10.1007/s00464-020-07621-5 (PMC8057995; doi:10.1007/s00464-020-07621-5)
Supplement: Supplementary file 3 — Supplementary file3 (PDF 143 kb) [file 464_2020_7621_MOESM3_ESM.pdf]

Supplementary File 3: Score items excluded in the Delphi Procedure

Excluded Outcomes (*In italic: specific reason for exclusion if applicable)*

Small Bowel Obstruction:

- Conservative treatment (i.a. nasogastric tube decompression, intravenous fluids therapy)
  Alternative options including time criteria were selected as most appropriate option and included with consensus.

Difficulties at reoperation:

- No difficulties at reoperation
  Alternative option 'reoperation without adhesiolysis' was selected as most appropriate option and included with consensus.
- Limited adhesiolysis (adhesiolysis <30min or adhesiolysis without injuries)
  Alternative option without time criteria were selected as most appropriate option and included with consensus
- Extensive adhesiolysis (Adhesiolysis >30min or Serosal bowel injuries)
  Alternative option without time criteria were selected as most appropriate option and included with consensus

Chronic Abdominal Pain:

- Intermittent or continuous chronic abdominal/pelvic pain during 6 months
- Intermittent or continuous chronic abdominal/pelvic pain during 12 months (or more)
- Chronic abdominal/pelvic pain with (deep) dyspareunia, dysuria or dyschezia
- Use of narcotics
- Surgical treatment for pain
  Reason for exclusion of outcomes listed above: Outcomes describing percentage of inability due to pain were preferred and included with consensus.

Female Infertility:

- Failure to conceive 1 year (Unprotected intercourse without conception 1 year)

Excluded Weight Factors

Small Bowel Obstruction:

- Small bowel obstruction, not further specified

- History of abdominal or pelvic surgery

Difficulties At Reoperation:

- Difficulties at reoperation, not further specified
- Adhesions found preoperatively on specialized imaging (cineMRI, visceral slide ultrasound)

Chronic Abdominal Pain:

- Chronic abdominal or pelvic pain, not further specified
- In women: non-cyclic pain
- Chronic pain in presence of other gastrointestinal complaints
- Psychiatric illness and psychological disturbances excluded or screened for
- Reduction in pain after adhesiolysis, followed by return of pain

Female infertility:

- Alternative explanation for female infertility at fertility investigation
- Female infertility, not further specified
- History of abdominal or pelvic surgery
- Abnormalities at fertility investigation (not further specified)

Supplementary File related to: "Clinical Adhesion Score (CLAS): Development of a novel clinical score for adhesion-related complications in abdominal and pelvic surgery"

Elisabeth Jacomine Lier[1] , Barend A.W. van den Beukel[1] , Larsa Gawria[1] , Prof. Dr. Philip J. van der Wees[2] , Leontine van den Hil[3], Dr. Nicole D. Bouvy[3,4], Prof. Dr. Ying Cheong[5,6] , Prof. Dr. Rudy-Leon de Wilde[7] , CLAS Collaboration, Prof. Dr. Harry van Goor[1], Dr. Martijn W.J. Stommel[1], Dr. Richard P.G. ten Broek[1]

Corresponding Author:
Janienke Lier, Department of Surgery, Radboud university medical center, Nijmegen, The Netherlands
Email: janienkelier@gmail.com
